# Zinc Sulfate Stimulates Osteogenic Phenotypes in Periosteum-Derived Cells and Co-Cultures of Periosteum-Derived Cells and THP-1 Cells

**DOI:** 10.3390/life11050410

**Published:** 2021-04-30

**Authors:** Jin-Ho Park, Su A Park, Young-Hoon Kang, So Myeong Hwa, Eun-Byeol Koh, Sun-Chul Hwang, Se Heang Oh, June-Ho Byun

**Affiliations:** 1 Department of Oral and Maxillofacial Surgery, Institute of Health Sciences, School of Medicine, Gyeongsang National University, Gyeongsang National University Hospital, Jinju 52727, Korea; jinho.park@gnu.ac.kr (J.-H.P.); omfs00@gnu.ac.kr (Y.-H.K.); hwasomyeong@gnu.ac.kr (S.M.H.); dmsquf0815@gnu.ac.kr (E.-B.K.); 2 Department of Convergence Medical Science, Gyeongsang National University, Jinju 52727, Korea; 3 Department of Nature-Inspired Nanoconvergence Systems, Korea Institute of Machinery and Materials, Deageon 34103, Korea; psa@kimm.re.kr; 4 Department of Orthopaedic Surgery, Institute of Health Sciences, School of Medicine, Gyeongsang National University, Jinju 52727, Korea; hscspine@gnu.ac.kr; 5 Department of Nanobiomedical Science, Dankook University, Cheonan 31116, Korea

**Keywords:** zinc sulfate, periosteum-derived cells, osteoblastic and osteoclastic differentiation, RANKL/OPG ratio

## Abstract

Coupling between osteoblast-mediated bone formation and osteoclast-mediated bone resorption maintains both mechanical integrity and mineral homeostasis. Zinc is required for the formation, mineralization, growth, and maintenance of bones. We examined the effects of zinc sulfate on osteoblastic differentiation of human periosteum-derived cells (hPDCs) and osteoclastic differentiation of THP-1 cells. Zinc sulfate enhanced the osteoblastic differentiation of hPDCs; however, it did not affect the osteoclastic differentiation of THP-1 cells. The levels of extracellular signaling-related kinase (ERK) were strongly increased during osteoblastic differentiation in zinc sulfate-treated hPDCs, compared with other mitogen-activated protein kinases (MAPKs). Zinc sulfate also promoted osteogenesis in hPDCs and THP-1 cells co-cultured with the ratio of one osteoclast to one osteoblast, as indicated by alkaline phosphatase levels, mineralization, and cellular calcium contents. In addition, the receptor activator of nuclear factor kappa B ligand (RANKL)/osteoprotegerin (OPG) ratio was decreased in the zinc sulfate-treated co-cultures. Our results suggest that zinc sulfate enhances osteogenesis directly by promoting osteoblastic differentiation and osteogenic activities in osteoblasts and indirectly by inhibiting osteoclastic bone resorption through a reduced RANKL/OPG ratio in co-cultured osteoblasts and osteoclasts.

## 1. Introduction

Bone remodeling is highly regulated to maintain a balance between osteoblast-mediated and osteoclast-mediated bone resorption. An imbalance of those tightly coupled processes can cause diseases such as osteoporosis. During bone turnover and remodeling, osteoblasts are closely associated with other cell types within bones, including osteoclasts [1,2].

Substantial progress has been made in the use of mesenchymal stem cell (MSC)-based therapeutic approaches to repair damaged, malfunctioning, or aging tissues. Osteogenic cells that have capacity for inducing bone formation in vivo can be derived from different human sources. The periosteum can be used as a good source of osteogenic cells to exert therapeutic efficacy. We previously examined that human periosteum-derived cells (*h*PDCs) express MSC markers and have differentiation potential toward adipocytes, osteocytes, and chondrocytes [3,4,5,6]. Hence, we refer to *h*PDCs as human periosteum-derived MSCs. Treatment of MSCs with growth factors or trace minerals can promote osteogenic differentiation of MSCs, increasing the likelihood that MSCs can be used for bone tissue repair [3,4,7,8].

Zinc is an essential element obtained through the diet that positively affects bone health and recovery of the bone architecture. Dietary zinc deficiency has been associated with changes in skeletal systems, including insufficient bone mineralization, skeletal malformation, and menopause-related bone loss. Although several forms of zinc are available as dietary supplements, zinc sulfate administered orally or parenterally is the most common form of dietary zinc supplement [9,10,11].

Zinc stimulates osteoblast differentiation and mineralization and inhibits osteoclast formation in vitro [12,13]. The presence of zinc in tissue culture media or substratum increases the expression of osteoblast-related genes; however, to our knowledge, its effects on osteoclast differentiation are not well characterized [12,13,14,15]. Moreover, the effects of zinc on osteogenesis in direct co-cultures of osteoblasts and osteoclasts that mimic the in vivo bone environment have not been well studied. Mitogen-activated protein kinases (MAPKs), including extracellular signaling-related kinase (ERK), c-Jun N-terminal kinase (JNK), and p38 MAPK, have been found to upregulate the expression of genes related to osteogenesis in several types of osteoprecursor cells [16,17,18]. However, the role of zinc in each MAPK signaling pathway during the differentiation of osteoblastic cells remains largely unknown.

We aimed to determine whether zinc sulfate affects the osteoblastic and osteoclastic phenotypes of *h*PDCs and THP-1 cells, respectively, during in vitro differentiation. We also investigated the effects of zinc sulfate on three MAPK signaling pathways (ERK, JNK, and p38 MAPK) during *h*PDC osteoblastogenesis. In addition, we examined the effects of zinc sulfate on osteogenesis co-cultures of *h*PDCs and THP-1 cells.

## 2. Materials and Methods

### 2.1. Culture and Differentiation of hPDCs

We harvested the periosteum from the mandibles of patients during extraction of impacted lower third molars at Gyeongsang National University Hospital. All periosteal tissues were obtained according to a protocol approved by the institutional review board (IRB) of our institution (approval no. GNUHIRB 2014-05-012). We isolated *h*PDCs from the harvested explants as previously described [6,7]. After 3–5 passages, we induced osteoblast differentiation by seeding the cells at a density of 3 × 10^4^ cells/well in osteogenic induction medium composed of DMEM supplemented with 10% FBS, 50 μg/mL L-ascorbic acid 2-phosphate, 10 nM dexamethasone, and 10 mM β-glycerophosphate. The media were changed every 2–3 days.

### 2.2. Measurement of hPDC Viability in the Presence of Zinc Suflate

To investigate the effects of zinc sulfate on the viability of *h*PDCs, we seeded *h*PDCs at a density of 3 × 10^4^ cells/well on 24-well plates in DMEM and incubated them for 24 h. Then, we treated the cells with 0, 1, 10, 100, or 1000 μM zinc sulfate (Sigma-Aldrich, St. Louis, MO, USA) for 24 h. The viability of the *h*PDCs was assayed using the Cell Counting Kit (CCK)-8 (Dojindo, Kumamato, Japan), using a previously published method [5,6].

### 2.3. Evaluation of Osteoblastic Differentiation of hPDCs Treated with Zinc Sulfate

To evaluate the effects of zinc sulfate on the osteoblastic differentiation of *h*PDCs, we treated periosteal cells that were previously cultured in osteogenic induction medium with 0, 1, 10, or 100 μM zinc sulfate and assessed the resulting phenotypes as previously described [5,6]. ALPs are considered to be relatively early markers of osteoblast differentiation, whereas the secretion of osteocalcin, matrix mineralization, and calcium contents are associated with the late phase of osteoblast differentiation [5,6]. We changed the medium every 2–3 days and added zinc sulfate at each change of the medium. We performed alkaline phosphatase (ALP) staining and activity assays on day 7 after the start of the zinc sulfate treatment. Fourteen days after the start of the zinc sulfate treatment, we determined alizarin red S staining and calcium contents of the cells.

### 2.4. Measurement of MAPK Phosphorylation in hPDCs

We examined the roles of MAPK signaling pathways on the osteogenic effects of zinc sulfate by Western blot analysis. To extract all proteins, hPDCs were placed in RIPA buffer (Cell Signaling Technology, Danvers, MA, USA) containing a protease and phosphatase inhibitor cocktail. After 20 min, the cell pellets were sonicated and centrifuged, and the supernatant was resolved by sodium dodecyl sulfate-polyacrylamide gel electrophoresis (SDS-PAGE), followed by the electrophoretic transfer onto a polyvinylidene difluoride membrane (Millipore, Burlington, MA, USA). We probed the membranes with primary antibodies against ERK, phospho-ERK, JNK, phospho-JNK, p38, and phospho-p38 (1:1000, all from Cell Signaling Technology) to analyze MAPK signaling specifically activated in *h*PDCs after zinc sulfate treatment; β-actin (1:1000, Cell Signaling Technology) was used as a positive control. Densitometry was performed using ImageJ software to quantify Western blot bands (Supplementary Materials).

### 2.5. Culture and Osteoclasitc Differentiation of THP-1 Cells

We established an osteoclast culture system to examine the effects of zinc sulfate on osteoclastic activities. First, we grew human THP-1 monocytic leukemia cells (ATCC, Manassas, VA, USA) in RPMI-1640 medium (Thermo Fisher Scientific, Waltham, MA, USA) supplemented with 10% FBS and 0.05 mM 2-mercaptoethanol (Thermo Fisher Scientific). The cells were incubated with 100 ng/mL phorbol 12-myristate 13-acetate (PMA, PeproTech, Rocky Hill, NJ, USA) for cell adherence for 3 days. Then, to induce osteoclastic differentiation, we cultured the cells at a density of 5 × 10^4^ cells/well on 24-well plates in osteoclastic induction medium composed of RPMI-1640 medium supplemented with 10% FBS, 0.05 mM 2-mercaptoethanol, 100 ng/mL PMA, 50 ng/mL macrophage colony-stimulating factor (M-CSF; R&D Systems, Minneapolis, MN, USA), and 50 ng/mL receptor activator of nuclear factor kappa B ligand (RANKL; R&D Systems). We changed the medium every 2–3 days.

### 2.6. Measurement of Effects of Zinc Sulfate on Osteoclast Differentiation

To investigate the effects of zinc sulfate on osteoclastic differentiation of THP-1 cells, we induced osteoclastic differentiation in the presence of 100 μM zinc sulfate (Sigma-Aldrich). Tartrate-resistant acid phosphatase (TRAP) activity is considered an important marker that is specifically produced by osteoclasts, so we used TRAP staining to assess the osteoclastogenic phenotypes of the cells on days 7 and 14 of osteoclastic induction [19]. We performed the TRAP staining using a TRAP staining kit according to the manufacturer’s protocol (Kamiya Biomedical Company, Tukwila, WA, USA). TRAP-positive multinucleated cells containing three or more nuclei were counted as osteoclasts.

In addition, on days 5, 10, and 15 of culture, we examined the expression of osteoclast-related genes (nuclear factor of activated T cells (NFATc1), calcitonin receptor (CTR), and cathepsin K) using Western blot analysis (all from Santa Cruz Biotechnology, Dallas, CA, USA).

### 2.7. Direct Co-Culture of hPDCs and THP-1 Cells and Effects of Zinc Sulfate on Osteoblastic Phenotypes

A direct co-culture model with osteoblasts and osteoclasts was established to mimic the in vivo environment in bones. We co-cultured the *h*PDCs from passage 1 and THP-1 cells on 24-well plates at a density of 5 × 10^4^ cells/well, in an equal volume mixture of osteogenic induction medium and osteoclastic induction medium with 0, 1, 10, or 100 μM zinc sulfate. To evaluate osteogenesis, we performed ALP staining and activity assays after 2 weeks of co-culture. We performed alizarin red S staining for mineralization and measured the calcium contents of the cells on day 21 of co-culture.

### 2.8. Measurement of RANKL and OPG Levels and the RANKL/OPG Ratio in hPDC and THP-1 Co-Cultures

To determine the effects of zinc sulfate on osteoclastic activity in the co-cultures of *h*PDCs and THP-1 cells treated with 100 μM zinc sulfate, we measured the RANKL and osteoprotegerin (OPG) protein levels and determined the RANKL/OPG ratio by Western blot using primary antibodies against RANKL and OPG (all from Abcam, Cambridge, MA, USA).

### 2.9. Statistical Analysis

All experiments were performed at least three independent times. The data obtained from designed experiments were expressed as mean with standard deviation. We used one-way analysis of variance (ANOVA) with Tukey’s multiple comparisons (SPSS Statistics 22 software, IBM, New York, NY, USA) for all statistical analyses. We considered comparisons with *p* < 0.05 to be statistically significant.

## 3. Results

### 3.1. Zinc Sulfate Promoted Osteoblast Differentiation

**Figure 1:**
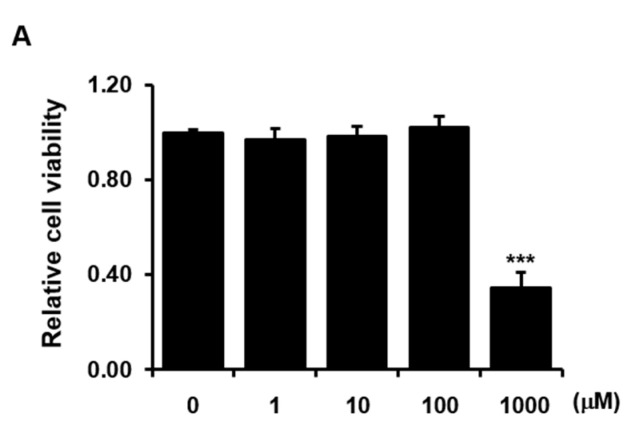

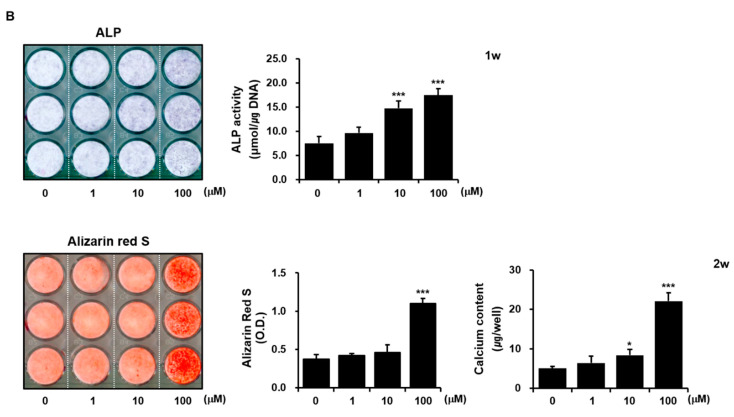
The effects of zinc sulfate on the viability and osteoblastic differentiation of *h*PDCs. Effects of zinc sulfate treatment on (**A**) viability and (**B**) bioactivity of ALP, alizarin red-positive mineralization, and calcium levels in *h*PDCs. * *p* < 0.05, *** *p* < 0.001.

Culture with 1000 μM zinc sulfate significantly reduced the viability of *h*PDCs, whereas culture with lower concentrations of zinc sulfate had no effect on *h*PDC viability compared with that in the absence of zinc sulfate (Figure 1A). Therefore, we used zinc sulfate at concentrations less than or equal to 100 μM in our subsequent experiments to test its other phenotypic effects.

Treatment with 1 µM zinc sulfate had no effect on the histochemical expression and activity of ALP in *h*PDCs, whereas treatment with 10 µM or 100 µM zinc sulfate markedly increased ALP detection and activity in *h*PDCs. Similarly, treatment with 1 µM zinc sulfate did not affect the calcium content of the cells, whereas treatment with 10 µM or 100 µM zinc sulfate resulted in increased calcium contents. Furthermore, zinc sulfate at concentrations less than 100 µM had no effect on alizarin red-positive mineralization in *h*PDCs, whereas 100 µM zinc sulfate clearly increased mineralization compared with that in untreated control cells (Figure 1B). These results suggest that zinc sulfate directly exerts positive effects on osteoblastic differentiation of *h*PDCs by promoting ALP activity and mineralization.

### 3.2. ERK Phosphorylation Was Strongly Induced after Zinc Sulfate Treatment in hPDCs

Three types of MAPK control key transcriptional events that mediate osteoblastic differentiation of osteoprecursor cells. A variety of extracellular stimuli, including environmental stress, growth factors, and cytokines, can activate MAPK pathways through serial phosphorylation; however, the roles of MAPKs in zinc sulfate-induced osteoblastic differentiation have not been well characterized [16,17,18,20,21]. As 100 µM zinc sulfate had clearly favorable effects on the osteoblastic differentiation of *h*PDCs, we analyzed the roles of the ERK, JNK, and p53 MAPK signaling pathways in the zinc sulfate-mediated enhancement of *h*PDC osteogenic potential.

**Figure 2:**
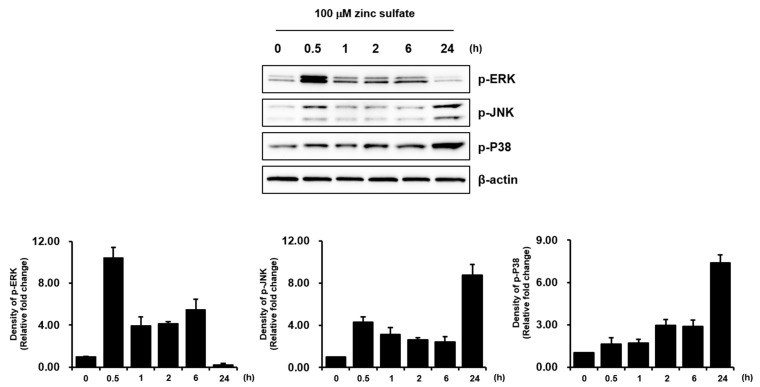
ERK signaling was strongly activated after zinc sulfate treatment in *h*PDCs.

Treatment with 100 µM zinc sulfate stimulated ERK and JNK phosphorylation but not p38 MAPK phosphorylation (Figure 2). Densitometry data generated for Western blot bands showed that ERK phosphorylation was induced more strongly than JNK phosphorylation in the zinc sulfate-treated *h*PDCs at 0.5 h (Figure 2). These results suggest that activation of MAPK signaling, and particularly ERK signaling, is involved in the zinc sulfate-mediated stimulation of *h*PDC osteoblastic differentiation.

### 3.3. Zinc Sulfate Did Not Affect the Osteoclastic Differentiation of THP-1 cells

**Figure 3:**
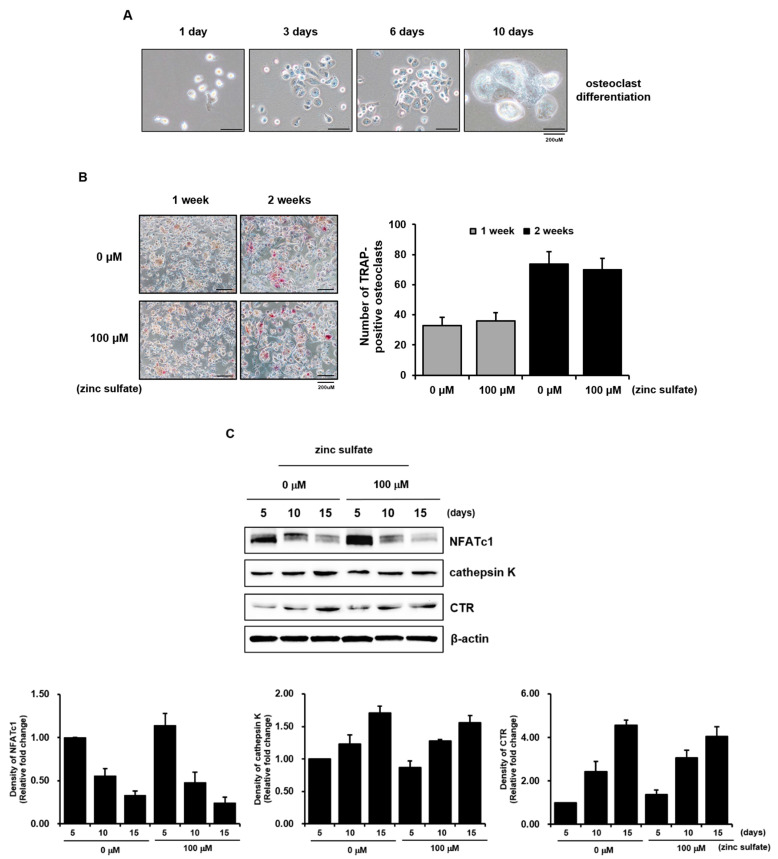
Effects of zinc sulfate on osteoclastic differentiation of THP-1 cells. (**A**) Multinucleated giant cells were observed during THP-1 cell differentiation. (**B**) Treatment with 100 μM zinc sulfate did not have significant effects on TRAP expression and TRAP-positive numbers of THP-1 cells. (**C**) Treatment with 100 μM zinc sulfate did not affect the expression of osteoclast-related genes.

Although zinc is an important trace element required for bone formation, and zinc sulfate shows its effects on the skeleton through various mechanisms involving both activation of osteoblastic bone formation and inhibition of osteoclastic bone resorption, evidence regarding the effects of zinc sulfate on osteoclastic differentiation is limited [12,13,14,15]. Therefore, we investigated the effects of zinc sulfate on osteoclastic differentiation in vitro using THP-1 cells. Osteoclasts are large multinucleated cells formed from the fusion of mononuclear precursor cells belonging to the monocyte/macrophage lineage. Osteoclastogenesis is a multi-complex process including osteoclast progenitor commitment, differentiation and fusion to multinucleated pre-osteoclasts which do not resorb bone, and activation into functional bone-resorbing osteoclasts [19]. When we induced osteoclastic differentiation in THP-1 cells, we observed large, round, multinuclear cells, which we took to be generated by fusion (Figure 3A). The multinuclear cells stained positively for TRAP and expressed several osteoclast-related genes (NFATc1, CTR, and cathepsin K; Figure 3B,C). These results show that THP-1 cells can be induced to differentiate into active osteoclasts, as demonstrated by previous studies [22,23,24].

As 100 μM zinc sulfate had clearly favorable effects on the osteoblastic differentiation of *h*PDCs, we next examined the effects of 100 μM zinc sulfate on the osteoclastic differentiation of THP-1 cells. Treatment with 100 μM zinc sulfate did not clearly affect the number of TRAP-positive THP-1 cells or the expression of NFATc1, cathepsin K, and CTR. The relative expression ratio by densitometric measurements of Western blots also showed that zinc sulfate did not have significant effects on the osteoclastic phenotypes of the THP-1 cells (Figure 3B,C). Although zinc has been reported to have a profound effect on bone turnover, activating bone formation and suppressing bone resorption, our results indicate that zinc sulfate did not directly affect the osteoclastic activities of THP- 1 cells in vitro.

### 3.4. Zinc Sulfate Promoted Osteogenic Phenotypes in Co-Cultures of hPDCs and THP-1 Cells

**Figure 4:**
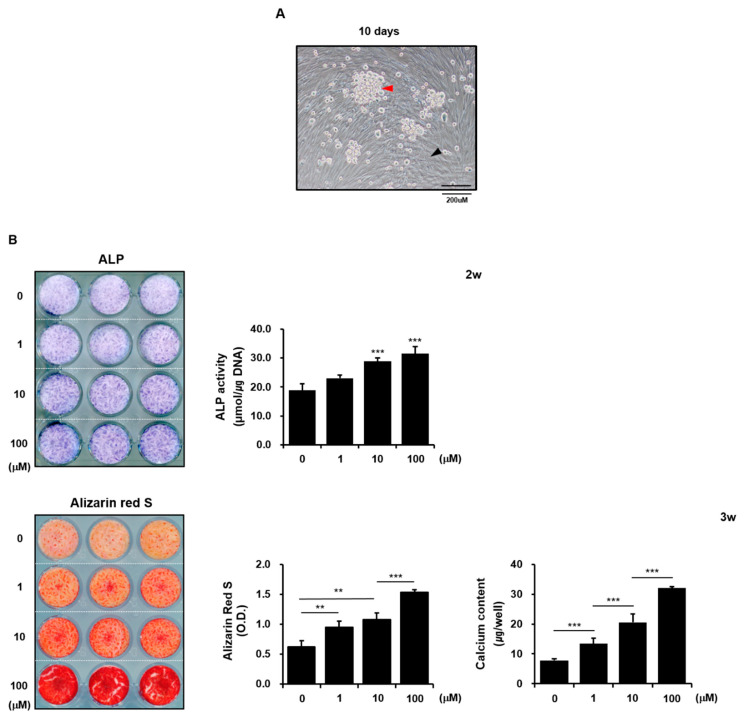
Effects of zinc sulfate on osteogenesis-related phenotypes in co-cultures of *h*PDCs and THP-1 cells. (**A**) Representative image of co-cultured *h*PDCs and THP-1 cells after 10 days of culture. (**B**) Effects of zinc sulfate treatment on the expression and bioactivity of ALP, mineralization, and calcium levels in co-cultures of *h*PDCs and THP-1 cells. Black arrow, osteoblasts; red arrow, multinucleated osteoclasts. ** *p* < 0.01. *** *p* < 0.001.

To model the effects of zinc sulfate on bone formation potential in vivo, we used *h*PDCs and THP-1 cells to establish direct co-cultures of osteoblasts and osteoclasts. Although limited research exists regarding the optimization of the osteoblast/osteoclast ratio in direct co-cultures, we chose the ratio of 1:1 osteoblast/osteoclast based on a previously published method [25]. The co-cultures of *h*PDCs and THP-1 cells grew well in an equal volume mixture of osteogenic induction medium and osteoclastic induction medium. The *h*PDCs exhibited a multi-layered cuboidal appearance, while the THP-1 cells displayed a large multinucleated morphology due to fusion (Figure 4A).

Similar to the effects of zinc sulfate on ALP expression in *h*PDCs, 10 µM or 100 µM zinc sulfate clearly increased the histochemical expression and bioactivity of ALP in the co-cultured cells after 2 weeks of culture. Zinc sulfate also stimulated the alizarin red-positive mineralized nodule formation and calcium contents of the co-cultured cells in a concentration-dependent manner after 3 weeks of culture (Figure 4B). These results demonstrate that zinc sulfate can be beneficial for the expression of osteoblastic phenotypes in *h*PDCs and THP-1 cells co-cultured.

### 3.5. Effects of Zinc Sulfate on the Levels of RANKL, OPG, and the RANKL/OPG Ratio in Co-Culture of hPDCs and THP-1 Cells

**Figure 5:**
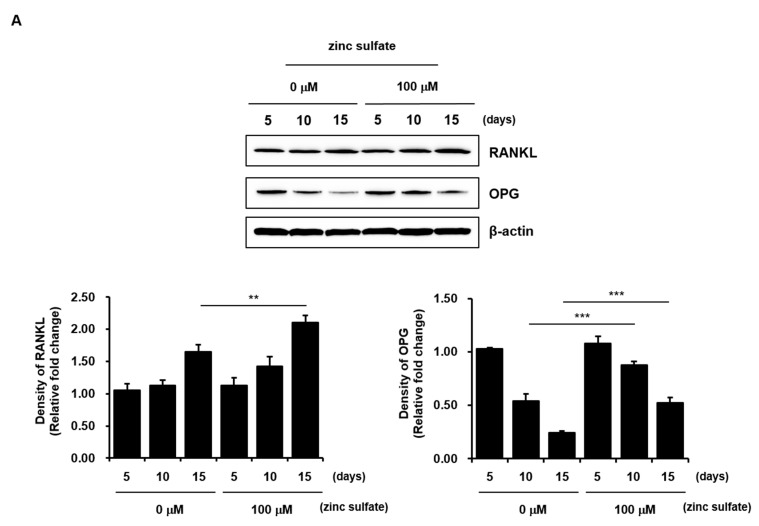

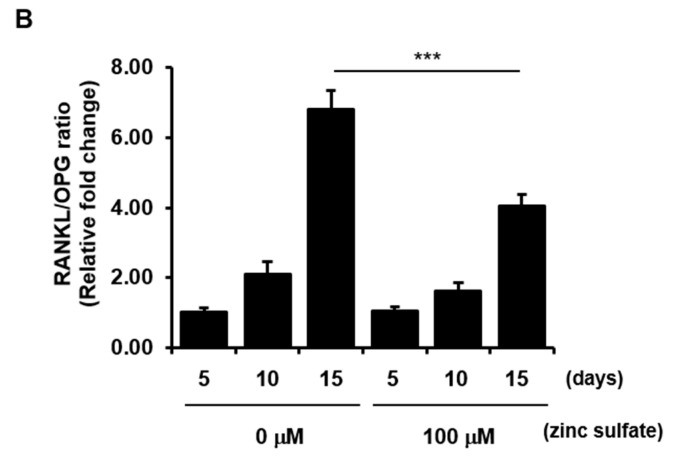
Effects of zinc sulfate on the levels of RANKL and OPG and the RANKL/OPG ratio in co-cultured *h*PDCs and THP-1 cells. (**A**) Protein levels of RANKL and OPG in co-cultured *h*PDCs and THP-1 cells treated with or without zinc sulfate after 5, 10, and 15 days of culture. (**B**) Measurement of the RANK/OPG ratio in co-cultured cells treated with or without zinc sulfate after 5, 10, and 15 days in culture. ** *p* < 0.01. *** *p* < 0.001.

RANKL and OPG act as positive and negative mediators of osteoclastogenesis, respectively, and are considered to affect bone resorption. The relative ratio of RANKL/OPG regulates the formation and activation of osteoclasts [19,26,27]. However, the effects of zinc on the levels of RANKL and OPG have not yet been elucidated in direct co-cultures of osteoblasts and osteoclasts. Therefore, although zinc sulfate did not directly affect the osteoclastic activities of THP-1 cells under the in vitro cell culture conditions used in our study, we examined the levels of RANKL and OPG and the RANKL/OPG ratio after treatment of co-cultured *h*PDCs and THP-1 cells with 100 µM zinc sulfate. The zinc sulfate treatment did not alter the level of RANKL in the co-cultures after 5 or 10 days of culture; however, it significantly increased the level of RANKL after 15 days of culture. Similarly, although the zinc sulfate treatment did not affect the level of OPG in the co-cultures after 5 days of culture, it increased the level of OPG after 10 and 15 days of culture (Figure 5A). In addition, the RANKL/OPG ratio, an important indicator of bone mass in both pathological and normal states, was dramatically decreased after 15 days of culture in the 100 µM zinc sulfate-treated co-cultured cells compared with that in untreated co-cultures (Figure 5B). These results suggest that zinc sulfate can indirectly contribute to osteogenesis by reducing osteoclastogenesis through a decrease in the RANKL/OPG ratio.

## 4. Discussion

As a cofactor of enzymes that support DNA, RNA, and protein synthesis, zinc has a positive effect on bone formation and mineralization [12,13,14,15,28]. Although zinc is required for normal bone homeostasis, the mechanisms by which it influences bone formation and bone resorption are not fully clarified, especially with regard to its effects on osteoclast activity.

We investigated the effects of zinc sulfate on osteoblastic activity in *h*PDCs. Our previous study showed that *h*PDCs differentiate into active osteoblastic cells capable of causing mineralization of a matrix. Zinc sulfate at concentrations less than 10 µM did not affect mineralization or the histochemical expression and bioactivity of ALP in *h*PDCs, whereas concentrations of 10 µM and 100 µM clearly affected all of those factors. These results suggest that zinc sulfate directly stimulates the in vitro osteoblastic differentiation of *h*PDCs.

MAPK pathways function as important regulators in osteoblastic differentiation of osteoprecursor cells through serial phosphorylation [16,17,18]. Although zinc enhances osteoblastic differentiation in human MSCs via activation of several pathways, the roles of each MAPK signaling pathway in the zinc sulfate-mediated osteoblastic differentiation of *h*PDCs remain unclear. We found that zinc sulfate treatment of *h*PDCs promoted ERK and JNK phosphorylation but had little effect on p38 MAPK phosphorylation. The effects of the zinc sulfate treatment on ERK phosphorylation were stronger than those on any of the other MAPKs tested.

Zinc can negatively affect osteoclast function while promoting osteoblast function, which is consistent with a favorable role of zinc in bone homeostasis and development [12,13,14,15]. However, we found that zinc sulfate did not directly affect the osteoclastic activities of THP-1 cells. Although further study is needed to elucidate the role of zinc sulfate in the osteoclastic differentiation of THP-1 cells, our findings suggest that zinc sulfate is more important for enhancing osteoblastogenesis than for reducing osteoclastogenesis.

We next investigated the effects of zinc sulfate on in vitro osteogenesis in co-cultures of *h*PDCs and THP-1 cells. Co-culture systems are valuable for assessment of in vitro bone formation and resorption, as they mimic the in vivo environment. Similar to its effects on monocultured *h*PDCs, zinc sulfate stimulated osteogenic phenotypes in co-cultured *h*PDCs and THP-1 cells with the ratio of one osteoclast to one osteoblast.

Osteoclastogenesis is regulated by two essential cytokines, M-CSF and RANKL, secreted from stromal cells and osteoblasts. M-CSF stimulates the proliferation and survival of osteoclast precursors by interacting with its receptor, colony-stimulating factor 1 receptor (CSF1R/c-FMS), on osteoclast precursor cells, leading to expression of receptor activator of NF-κB (RANK) and activation of the RANKL/RANK signaling pathways. RANKL is a type II homotrimeric transmembrane protein that, when released, binds to its receptor RANK, which is expressed on the surfaces of osteoclast precursors and functions as a key signal for osteoclast differentiation and activation. Therefore, osteoclastogenesis requires osteoclast progenitor commitment, M-CSF-mediated osteoclast precursor proliferation, and RANKL-mediated osteoclast differentiation. RANK-RANKL binding activates the NF-κB pathway, resulting in the upregulation of the transcription factor NFATc1 via orchestrated signaling of activated activator protein-1 (AP-1) and co-stimulatory signal-mediated intracellular Ca^2+^ oscillation. Finally, the osteoclastogenic genes transcribed by activated NFATc1 regulate the multinucleation and bone resorption function of osteoclasts. OPG is a soluble receptor of RANK that functions as a competitive inhibitor of RANKL, reducing osteoclastogenesis and bone resorption [29,30,31,32,33,34,35,36]. Thus, the RANKL/OPG ratio in bone is pivotal in the regulation of osteoclast differentiation and affects the coupling between bone formation and resorption. We found that although 100 µM zinc sulfate increased the levels of both RANKL and OPG in co-cultured *h*PDCs and THP-1 cells after 15 days of culture, the RANKL/OPG ratio was markedly decreased in the zinc sulfate-treated co-cultured cells.

Unfortunately, in the present study, the effects of zinc sulfate on osteoclastic activities in co-cultured *h*PDCs and THP-1 cells were not evaluated. However, considering that the direct co-culture system using osteoblasts and osteoclasts reflects part of the complex process of bone homeostasis that takes place in vivo, combined with the direct effects of zinc sulfate on the enhanced osteogenic differentiation of the *h*PDCs and the lack of effects of zinc sulfate on the osteoclast differentiation of THP-1 cells, our findings suggest that zinc sulfate can also indirectly promote osteogenesis in vivo and in co-cultured osteoblasts and osteoclasts in vitro by reducing osteoclastogenesis via a decrease in the RANKL/OPG ratio.

## Data Availability

Data are included in the text; raw data are available from the corresponding author.

## Supplementary Materials

The following are available online at https://www.mdpi.com/article/10.3390/life11050410/s1, Figure S1. Original Western Blot Figure.
