# Supplementary figures and images for: Zinc Sulfate Stimulates Osteogenic Phenotypes in Periosteum-Derived Cells and Co-Cultures of Periosteum-Derived Cells and THP-1 Cells
Source: Life (Basel). 2021 Apr 30;11(5):410. doi: 10.3390/life11050410 (PMC8144993; doi:10.3390/life11050410)

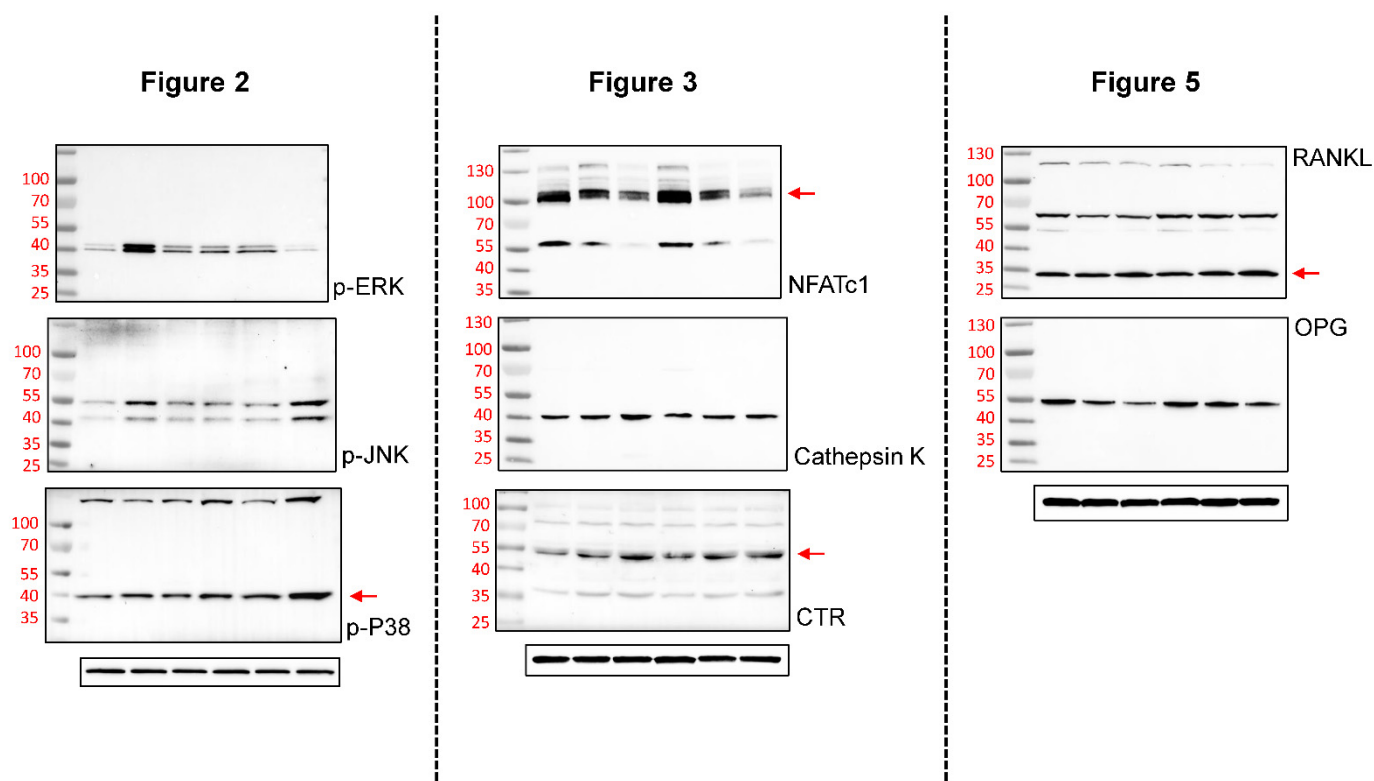

Figure S1. Original Western Blot Figure.

Supplement: Supplementary file 1 [file life-11-00410-s001.zip › life-1194786-supplementary.pdf]
